# Editorial Special Issue: “Advance Polymeric Materials: Synthesis, Properties and Applications”

**DOI:** 10.3390/ma15082934

**Published:** 2022-04-18

**Authors:** Manuela Zubitur

**Affiliations:** POLYMAT and Chemical and Environmental Engineering Department, Faculty of Engineering, University of the Basque Country UPV/EHU, 20018 Donostia-San Sebastián, Spain; manuela.zubitur@ehu.eus

As a consequence of their properties of lightness, strength, versatility, low toxicity, low cost and durability, the 20th century saw the beginning of the use of polymers as disruptive materials. They replaced traditional materials in automotive, medicinal and packaging applications, and made outstanding contributions to improving the general quality of life.

However, solutions created in the last century are redundant to many problems of the present day. Nowadays, the reputation of polymers has been overshadowed by increasing concerns of sustainability issues regarding their environmental impact.

Material researchers are tackling these problems with the hope to demonstrate that polymers can also be part of the solution, and a “material of the future”. The innovation of these advanced materials is focused on the incorporation of new functionalities and the improvement of their properties, without losing sight of sustainability.

In this context, polymer systems have surfaced as a way to produce new, sustainable, efficient, and versatile materials. Advanced polymer systems are characterized as combinations of materials with improved characteristics (mechanical strength, conductivity, biodegradability) or functionalities (self-healing, anti-fouling, controlled substance release) that give them certain properties, performance, or applications of great industrial interest. Therefore, the development of tailored polymer systems as copolymers, nanocomposites and blends will allow us to tackle technological challenges at the same time as addressing the global need for sustainability.

Blending two or more polymers is an effective way to overcome the drawbacks that limit the use of polymers, and to produce materials with enhanced properties. Specific properties can be achieved depending on the composition and preparation methods. In this sense, in order to widen the application of biopolymers to fields such as the automotive and electronic sectors, their performance must be substantially improved. This can be obtained through blending. For example, PLA is a biopolymer with relatively poor mechanical properties; blending this polymer with a flexible non-biodegradable (PE) or biodegradable (PCL) polymer leads to an enhancement of PLA toughness.

Blending environmentally friendly biopolymer-based materials from renewable natural sources (e.g., cellulose) with synthetic polymers (polystyrene) can help to reduce the use of fossil resources and increase the biodegradability of the material.

Nanocomposites are another type of polymer system where the addition of nanofillers improves polymer properties, such as mechanical performance, thermal stability, flammability, biodegradability, etc. Different type of nanofillers can be used, e.g., 1D (CNT, halloysite nanotubes), 2D (clay, graphene), and 3D (SiO_2_, TiO_2_…). The integration of nanofiller with the polymer matrix, in some cases, leads to a unique combination of properties (such as electrical, magnetic and optical) that meet the needs of advanced applications in the electronic and biomedical sectors.

Copolymers are another type of advanced polymer system (random, alternating, graft, block, or network); this process involves combining polymers with different thermal and mechanical properties, resulting in tuned materials that can used for different applications.

Another key factor to take into account is that emerging technologies, such as 3D printing, require new optimized polymeric materials. Therefore, the development of blends, nanocomposites and copolymers for 3D printing will offer new alternatives with lower environmental impact and enhanced properties.

Ultimately, a number of the challenges of the 21st century—such as those related to energy, healthcare, and sustainability—demand the development of advanced polymeric systems with specific properties and multifunctionality. These materials will lead to future technological innovations, and will contribute to building a better future in line with global Sustainable Development Goals (United Nations Agenda 2030).

This Special Issue, “Advanced Polymeric Materials: Synthesis, Properties, and Applications”, will include recent innovations and developments on a range of advanced polymer systems, including: polymer blends, nanocomposites and copolymers with properties such as self-healing, sensing, or switching; polymers with applications in coatings and textiles; and 3D printing and drug delivery systems.

As Guest Editor, it is my pleasure to invite contributions in the form of original research articles or reviews on this subject.

